# Maternal age and genome-wide failure of meiotic recombination are associated with triploid conceptions in humans

**DOI:** 10.1016/j.ajhg.2025.09.014

**Published:** 2025-10-14

**Authors:** Ludovica Picchetta, Christian Simon Ottolini, Xin Tao, Yiping Zhan, Vaidehi Jobanputra, Carlos Marin Vallejo, Francesca Mulas, Elvezia Maria Paraboschi, Maria José Escribá Pérez, Thomas Molinaro, Christine Whitehead, Pavan Gill, Emily Mounts, Dhruti Babariya, Laura Francesca Rienzi, Filippo Maria Ubaldi, Juan Antonio Garcia-Velasco, Antonio Pellicer, Shai Carmi, Eva R. Hoffmann, Antonio Capalbo

**Affiliations:** 1 Juno Genetics, Reproductive Genetics, Rome, Italy; 2 University of Teramo, Department of Department of Bioscience and Agro-Food and Environmental Technology, Teramo, Italy; 3 University College London Institute for Women’s Health, Department of Maternal and Fetal Medicine, London, UK; 4 Juno Genetics, Genetic Lab, Basking Ridge, NJ, USA; 5 Juno Genetics, Genetic Lab, Valencia, Spain; 6 IVI Foundation, Health Research Institute La Fe, Valencia, Spain; 7 IVIRMA Global Research Alliance, IVIRMA Valencia, Valencia, Spain; 8 IVIRMA Global Research Alliance, IVIRMA New Jersey, Basking Ridge, NJ, USA; 9 IVIRMA, Clinical Research, Basking Ridge, NJ, USA; 10 Juno Genetics, Genetic Lab, Oxford, UK; 11 IVIRMA Global Research Alliance, Genera, Clinica Valle Giulia, Rome, Italy; 12 Department of Biomolecular Sciences, University of Urbino "Carlo Bo," Urbino, Italy; 13 IVIRMA Global Research Alliance, IVIRMA, Madrid, Spain; 14 IVIRMA Global Research Alliance, IVIRMA, Rome, Italy; 15 Braun School of Public Health and Community Medicine, the Hebrew University of Jerusalem, Jerusalem, Israel; 16 DNRF Center for Chromosome Stability, Department of Cellular and Molecular Medicine, Faculty of Health and Medical Sciences, University of Copenhagen, Copenhagen, Denmark; 17 “G. D'Annunzio” University of Chieti-Pescara, Center for Advanced Studies and Technology CAST, Chieti, Italy

**Keywords:** triploidy, haploidy, genotyping, embryos, pregnancy loss, human development

## Abstract

Triploid and haploid conceptions are not viable and are a common occurrence in humans, where they account for 10% of all pregnancy losses. Despite the parent of origin being important in the etiology of the pregnancy, our knowledge of their causes is limited, especially at the point of conception. Using a dataset of 96,660 biopsies and a validation dataset of 44,324 from human blastocyst embryos generated by intracytoplasmic sperm injection, we estimate that 1.1% of human conceptions (*n* = 1,063) contain extra or missing chromosome sets in zygotes. In our cohort of intracytoplasmic-sperm-injection-derived embryos, where the risk of polyspermy is inherently lower compared to natural conception, we identify for the first time a maternal age effect, with a 1.046-per-year increased risk in triploidy/haploidy (*p* < 0.001). In 0.03% of couples, we identified three or more triploid/haploid embryos, suggesting a personal risk effect (*p* = 0.03). Genotype analysis of 41 triploid embryo biopsies and their parents shows that around one-third of maternal triploid conceptions originate in meiosis I and two-thirds in meiosis II. Seven of these embryos are inferred to have entirely failed to initiate meiotic recombination genome wide, a surprising finding suggesting that human oocytes with pervasive meiotic recombination failure that are formed during fetal development are capable of ovulation in adult life. Finally, we identify a type of genome-wide maternal isodiploidy (two maternal chromosome sets) in 0.05% of embryos (41/74,009). Collectively, our findings shed light on the biology of meiosis and the formation of human oocytes with the number of chromosome sets.

## Graphical abstract

**Figure:**
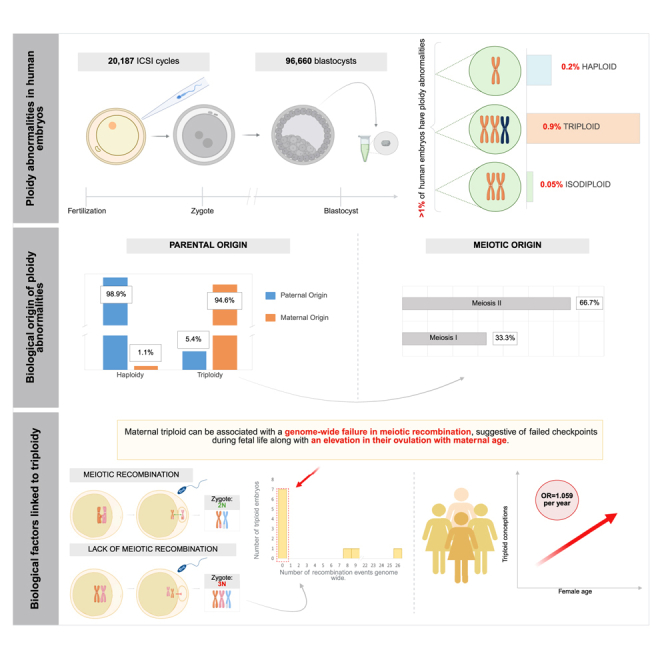

## Teaser

Ploidy abnormalities affect over 1% of human embryos. Haploidy typically results from absent sperm DNA, while triploidy mainly arises from maternal meiosis II errors. Triploidy also shows a linear maternal age effect and can be characterized by genome-wide recombination failure, indicative of defective meiotic checkpoints during fetal development.

## Introduction

Meiosis is a specialized cell division that reduces the genomic content by one-half, such that when an oocyte and sperm combine at fertilization, the diploid content is restored in the offspring. Failure to reduce the diploid content results in additional (polyploidy) or missing (haploidy) chromosome sets, a life-limiting condition in humans and other animals.^1^^,^^2^

In humans, 1% of conceptions are estimated to be triploid, and they make up 10% of all pregnancy losses, including during *in vitro* fertilization (IVF) treatment.^3^^,^^4^^,^^5^^,^^6^^,^^7^^,^^8^ As early as 1967, ploidy abnormalities were observed in tissue from pregnancy losses, chorionic carcinoma, and molar pregnancies, and they are recognized as one of the leading causes of pregnancy complications.^9^^,^^10^ Genetic analysis of fetal losses shows that about one-third of maternal-origin (digynic) triploidy originates in meiosis I (MI) (with the remaining in meiosis II [MII]) and that paternal (diandric) triploidy is mainly due to fertilization by two sperm or diploid single sperm.^11^ The parent of origin of the error is important clinically, as it is associated with distinct maternal risks, fetal abnormalities, and prenatal sonographic features.^12^^,^^13^^,^^14^

Triploidy was previously thought to be a random occurrence of abnormal fertilization.^3^^,^^15^^,^^16^^,^^17^^,^^18^ However, recent data from mouse models^19^ and genetic analysis of human polyploid conceptions suggest that a subset of individuals may be predisposed to abnormal ploidy conceptions.^20^^,^^21^^,^^22^^,^^23^ Epidemiological studies suggest no association with parental age or aberrant meiotic recombination,^24^^,^^25^ two factors strongly associated with aneuploidy in oocytes (where only one or a few chromosomes are affected). Triploidy is also common in other animals, such as horses^1^ and salmon.^2^

Our current knowledge of polyploid conceptions is limited to studies at stages of post-implantation fetal development, where selection against dysfunctional genomic constellations has already occurred. Therefore, we set out to leverage information from large datasets of preimplantation embryos that underwent genetic testing following intracytoplasmic sperm injection (ICSI) and extended the culture to the blastocyst stage of embryo development. This allowed us to detect a maternal age effect, genome-wide recombination failure, and a new type of isodiploidy that affects human oocytes, in a selected cohort of embryos where potentially confounding factors such as polyspermy could be excluded to take into account maternally contributing factors only.

## Subjects, material, and methods

### Study design

A retrospective cohort study was conducted by Juno Genetics, a clinical laboratory improvement amendment (CLIA)-certified genetic testing laboratory, assessing embryos from 62 referring reproductive medicine clinics in the United States between January 2020 and September 2023. Raw genetic data, collected as standard clinical practice, were leveraged for nonclinical analysis of ploidy-level abnormalities and any potential association with clinical/embryological parameters. To evaluate the impact of parental age, individuals were classified into five distinct age groups based on SART (Society for Assisted Reproductive Technologies) classification (web resources).

The first part of the study aimed at the comprehensive characterization of haploidy and triploidy. This analysis was conducted on a cohort drawn from a total of 96,660 trophectoderm biopsy samples taken from blastocyst-stage embryos derived from zygotes displaying two pronuclei (2PNs) following IVF via ICSI (20,187 cycles) (referred to as dataset A; Table S1). All embryos were subjected to clinical preimplantation genetic testing (PGT) for either chromosomal aneuploidy alone (PGT-A) due to a history of infertility (*n* = 87,064) or concurrent aneuploidy testing and testing for monogenic disorders (PGT-M) (*n* = 9596) due to a positive family history for a monogenic disorder. PGT was performed using a targeted next-generation sequencing (NGS) platform.^26^^,^^27^

To validate the findings on ploidy abnormalities and their relationship with clinical factors (e.g., maternal age), an independent cohort of 44,324 trophectoderm biopsy samples taken from blastocyst-stage embryos (referred to as dataset B; Table S1) was analyzed. Additionally, the association between maternal age and ploidy abnormality was tested using dataset C (Table S1), a cohort comprising 93,341 zygotes with corresponding annotation of the number of PNs displayed around 16 h post-IVF. Finally, a fourth dataset (referred to as dataset D; Table S1) consisted of 74,009 embryo biopsies with a 46,XX karyotype that were tested for aneuploidy by targeted NGS between January 2020 and September 2023. This dataset partially overlapped with dataset A (Table S1) and was used to investigate the incidence and origin of isodiploidy.

Institutional review board (IRB) approvals were obtained prior to research data analysis (Advarra Pro00074493; WIRB 1053149 and CEIM - Hospital Universitario y Politécnico de La Fe no. 2024-0405-1).

Informed consent was obtained from all individuals.

### Sequencing data analysis

PGT was performed clinically for embryo biopsy samples by first performing a targeted DNA amplification step, followed by NGS by PGTseq analysis (PGTseq Technology, Basking Ridge, New Jersey).^26^^,^^27^^,^^28^ In short, NextSeq500/550 or NovaSe6000 Mid and High Output Kit v.2.5 NGS-based PGT-A was used for TE (trophectoderm) biopsy chromosome copy-number (CN) analysis, with around 5,000 amplicons and single-nucleotide polymorphisms (SNPs) per sample. Proprietary PGTseq software was used for bioinformatics and automatic calls of chromosome CNs as previously described. Embryo ploidy abnormalities were clinically reported following a validated analytical pipeline of combined sequencing quantitation and genotyping allele ratio data analysis. Briefly, the allele ratio was obtained for each heterozygous SNP in each biopsy sample. In the instance of a balanced chromosome CN and unbalanced allele ratios (2:1) across the genome, indicative of 3 copies for each chromosome, the embryo was deemed triploid. If there was a total loss of heterozygosity across the genome, indicating the presence of only one allele with an allele ratio of 1:0 per chromosome, the embryo was deemed haploid.

A bespoke research bioinformatic pipeline was developed to more comprehensively analyze raw sequencing data from biopsy samples and to further investigate preimplantation embryo ploidy anomalies. BAM files were aligned against the GRCh37 human reference using BWA (Burrows Wheeler Aligner), and FREEBAYES v.1.3.2 was used with default settings to identify variants. Using vcf2tsv, genomic VCF files were converted to a tab-separated format suitable for downstream analysis. Customized allele frequency thresholds were established to generate discrete genotype calls based on the proportion of read counts mapped at the specific genomic locus as follows. Loci with an alternative allele frequency below 0.05 were defined as a homozygous reference, and loci with an allele frequency between 0.2 and 0.8 were deemed heterozygous, whereas loci with allele frequencies higher than 0.95 were called as a homozygous reference.

Variants were filtered according to the following criteria: (1) the variant was a bi-allelic SNP according to gnomAD v.2.1.1,^29^ allowing higher accuracy of the model; (2) the depth of coverage was greater than 20× (the average coverage was 360×, ranging from 0 to 10,104×), thus providing sufficient reads to estimate B-allele ratios; (3) the B-allele frequency (BAF) ranged between 5% and 95%; and (4) the variant mapped on an autosome.

### Parental and meiotic phase of origin of ploidy abnormalities using sex chromosome’s CN values

CN values of the sex chromosomes were used to infer the parental origin and meiotic origin of the ploidy abnormality in 1,063 haploid and triploid embryos.

This was accomplished in triploid embryos using a mathematical model based on a priory clinical diagnosis of a ploidy abnormality and the assumption that the error distribution between the two meiotic divisions (i.e., MI and MII) is the same in both sexes:

$$\begin{array}{c} N\left(\text{xxx}\right)=N\frac{1}{2}p\left(♀\right)\left[p\left(\text{MII}\right)+p\left(\text{MI}\right)\right]+N\frac{1}{2}p\left(♂\right)p\left(\text{MII}\right)\\ N\left(\text{xyy}\right)=N\frac{1}{2}p\left(♂\right)p\left(\text{MII}\right)\\ N\left(\text{xxy}\right)=N\frac{1}{2}p\left(♀\right)\left[p\left(\text{MII}\right)+p\left(\text{MI}\right)\right]+Np\left(♂\right)p\left(\text{MI}\right)\\ p\left(♂\right)=1-p\left(♀\right)\\ p\left(\text{MII}\right)=1-p\left(\text{MI}\right)\\ N=N\left(\text{xxx}\right)+N\left(\text{xxy}\right)+N\left(\text{xyy}\right) \end{array}\text{.}$$

When considering triploid embryos with chrX:chrY copy ratios of 3:0 [*N*(xxx)], 2:1 [*N*(xxy)], and 1:2 [*N*(xyy)], p(♀) is the probability that a triploid is due to a female error, p(♂) is the probability that a triploid is due to a male error, p(MI) is the probability that a triploid is due to an MI error, p(MII) is the probability that a triploid is due to an MII error, and *N* is the total number of triploid embryos.

Therefore, we obtained the following “method-of-moments” estimators:

$$\begin{array}{c} p\left(♀\right)=2\left[N\left(\text{xxx}\right)-N\left(\text{xyy}\right)\right]/N\\ p\left(♂\right)=1-2\left[N\left(\text{xxx}\right)-N\left(\text{xyy}\right)\right]/N\\ p\left(\text{MI}\right)=\left[N-2N\left(\text{xxx}\right)\right]\;/\;[N-2(N\left(\text{xxx}\right)-N\left(\text{xyy}\right)]\\ p\left(\text{MII}\right)=2N\left(\text{xyy}\right)\;/\;[N-2(N\left(\text{xxx}\right)-N\left(\text{xyy}\right)] \end{array}\text{.}$$

The same approach was also used on haploid samples, following the equation below. Due to the lack of genetic data from the parent in whom the error occurred, it was not possible to distinguish between MI and MII in this subset.

$$\begin{array}{c} N\left(x\right)=Np\left(♂\right)+N\frac{p\left(♀\right)}{2}\\ N\left(y\right)=N\frac{p\left(♀\right)}{2}\\ p\left(♂\right)=1-p\left(♀\right)\\ N=N\left(x\right)+N\left(y\right) \end{array}\text{.}$$

Therefore, we obtain

p(♀)=2N(y)/Nand

p(♂)=(N(x)−N(y))/N.

### Parental and meiotic phase of origin of ploidy abnormalities using genotyping data

Using the genotypic output of a subset of ploidy-abnormal embryos analyzed through concurrent PGT-A and PGT-M, a second independent and more comprehensive methodology (compared to sex chromosome CN analysis) was applied to calculate the parent and meiotic origins of the extra/missing haploid set of chromosomes in ploidy-abnormal embryos. In total, embryonic and parental genotyping data from 55 trios were analyzed, including 41 triploids and 14 haploids. To determine the parental origin (in both triploid and haploid abnormal embryos), the informative SNPs had to be opposite homozygous in the two parents. As an example, if the maternal genotype was “AA” and the paternal genotype was “BB” at the same locus, an occurrence of “ABB” for the embryo was defined as a paternal error, whereas “AAB” for the embryo was defined as a maternal error (determined according to BAFs around 0.33 and 0.66). The total counts of paternal errors (Sp) and maternal errors (Sm) were used to compute a “parental origin score,” defined as log(Sm/Sp). Positive values of this score denoted that the ploidy abnormality was due to a maternal error, while negative scores indicated that the ploidy abnormality was due to a paternal error.

To determine the meiotic phase of origin, the variant had to be within 5 cM of the centromere (to avoid dissociation of the SNP from the centromere due to meiotic recombination events) and one parent (the one in whom the error happened) had to be heterozygous (e.g., AB), while the other was homozygous (e.g., AA or BB). This information can be used to identify SNPs indicative of the presence of both parental homologs (BPHs) or the presence of a single parental homolog (SPH). Due to inherent genetic constraints, meiotic-phase analysis could only be performed on triploid samples. Similar to the parental origin score, a “meiotic origin score” was computed as log(S2/S1), with S1 and S2 representing the observed number of BPH SNPs and SPH SNPs, respectively. As an example, if the maternal genotype was “AB” and the paternal genotype was “AA” at the same locus, an occurrence of “AAB” for the embryo was defined as a BPH and thus an MI error, whereas “AAA or ABB” for the embryo was defined as an SPH and an MII error. Positive values of this score denoted an enrichment in SPH and an MII origin of the extra set of chromosomes, while negative scores indicated an MI origin of the extra set of chromosomes, with most SNPs being BPHs.

### Recombination analysis

Data from 41 triploid embryos with concurrent aneuploidy and monogenic disorder testing were used, along with the genotyping data from their parents, to estimate the rate of recombination.

For each embryo, autosomal SNPs were selected using the filtering parameters as previously described in the previous section. For each SNP, concordance with a BPH or SPH model was evaluated using probabilities according to Mendel’s first law. If the embryonic genotype was concordant with either the presence of a BPH or SPH, a concordance score of 0 was assigned. Conversely, if the SNP was not concordant with the model, a score of 1 was assigned. For each SNP, a delta of the BPH and SPH concordance score was calculated (0, 1, −1). Using a sliding-window approach from telomere to telomere for each chromosome, an average of the score was computed for each window of 3 consecutive SNPs. A switch of the average concordance score from 1 to −1 (or vice versa) was considered a marker of recombination due to evidence of a phase change from a BPH to an SPH (or vice versa). Therefore, this locus and the closest preceding SNP with an opposite average concordance score value were used as the ending and starting points of genomic windows in which recombination occurred (Table S5). The size of the sliding windows was chosen to account for embryo genotypes that could be attributed to both BPHs and SPHs and based on an estimation of the SNP genotyping error rate (0.42%) calculated as an average of inconsistent embryo genotypes according to the parental genotypes (Table S6). Therefore, the chance of observing three consecutive SNP genotyping errors was as low as 7 × 10^−8^ (0.0042^3^).

### Genome-wide uniparental isodisomy in diploid blastocysts

To determine the incidence of diploid embryos with the duplication of one and the concomitant loss of the other parental genome (isodiploidy), we used a dataset of 74,009 embryo biopsies with a 46,XX karyotype from aneuploidy testing by targeted NGS (dataset D, Table S1). While some samples may overlap with the first dataset of 96,660 biopsies, this dataset also includes additional clinical data obtained after the completion of the initial set of analysis on ploidy abnormalities.

A bespoke algorithm was developed to interrogate the overall fraction of heterozygous SNPs (total het fraction, including heterozygous SNPs mapping on autosomes and on chromosome X) and the pericentromeric fraction of heterozygous SNPs (pericentromeric het fraction within 10 Mb of centromeres) of autosomes and chromosome X. The heterozygous fraction was defined as the fraction of SNPs with a BAF between 0.1 and 0.9. Samples with a total het fraction lower than the average for all samples and with a statistically significant lower pericentromeric het fraction, compared to the average and the overall fractions of the sample itself, were marked as possible recombinant isodiploidy. Subsequently, each sample was manually checked to confirm that the partial genome-wide loss of heterozygosity was visible in the alternate allele fraction distribution in the aneuploidy testing plot. Genetic similarity via fingerprinting analysis was also performed to evaluate the genetic correlation between sibling embryos and, where available, parental DNA.

### Statistical methods

Association between ploidy abnormalities and maternal and/or paternal age was assessed by performing multivariate logistic regression. As a positive control, the same dataset was also analyzed to corroborate an association between advancing maternal age (grouped according to SART classification) and aneuploidy rates utilizing a chi-squared test. Additionally, an independent dataset of 93,341 oocytes from 15,851 cycles was used to assess age association with morphologically atypical PN patterns, which are believed to correlate with abnormal ploidy status (Table S1). Validation of the parental age correlation findings was performed by analyzing an independent dataset of 44,324 embryos utilizing the same statistical methodology (Table S1).

To study the recurrence of abnormalities, female and male individuals were first grouped into five categories according to age, as described above. For each age category, the recurrence of ploidy abnormalities in the same IVF cycle was investigated by taking, as a null model, a random permutation of the embryo ploidy level across all samples under analysis, ensuring that the null model had the same distribution of the number of embryos per cycle as in the original dataset. A *p* value was estimated for each observed recurrence in each age category as a cumulative probability from the corresponding null model. *p* values were considered statistically significant if *p* < 0.05 following Bonferroni correction.

Finally, a *t* test was used to test for correlation between recombination rates and the average aneuploidy rate per embryo.

### Validation of genotyping methods

Validation of the genetic findings was performed by analyzing the re-biopsies of 9 selected embryos (Table S7). The reproducibility of our methods was assessed by analyzing new biopsies with the same technology and PGT algorithms. Additionally, the meiotic origin of triploidy, the number of recombination events in triploid embryos, and the diagnosis of isodiploidy were confirmed by means of a SNP array (Infinium Global Screening Array-24 v.3.0, Illumina).

For the SNP-array data, SNPs heterozygous for one parent and homozygous for the other were considered informative (i.e., AB and AA or BB). Assuming from previous PGT results, all abnormalities were maternal in origin, so only maternal heterozygous SNPs were evaluated. Each embryonic SNP was assigned, in a binary fashion, a value of either “1,” corresponding to BPH/SPH (heterozygous SNP, which includes genotypes such as “AAB” or “ABB”), or “0,” corresponding to SPH (homozygous SNP). Evaluation of pericentromeric SNPs was performed to infer the meiotic phase of origin.

To identify the breakpoints of meiotic recombination in triploid embryos, genomic regions of BPHs and SPHs were defined using the moving average smoothing algorithm. A sliding window of 50 SNPs was applied from the centromere toward each telomere of each chromosome, and the average BPH and SPH SNP value was calculated within the window.

BPH was assigned if at least 90% of SNPs within the window were of value 1. Similarly, SPH was assigned if the SNPs within the window were equally distributed between 1 and 0 (threshold 50%). The assignment of a recombination breakpoint was made at the point of switch between BPH and SPH, defined by these thresholds.

To determine the statistical significance of obtaining the observed overlap between recombination breakpoints defined by SNP-array and PGT-derived windows of recombination, a permutation approach was adopted for each embryo. For each PGT-derived recombination window, we obtained a window of the same size but placed randomly on the same chromosome. This procedure was repeated 1,000 times, and the overlap of SNP-array points, with the randomly placed windows, was computed to obtain a null distribution. *p* values were estimated as cumulative probabilities of the observed overlap from the null distribution.

Seven out of seven triploid embryos and two out of two isodiploid embryos were confirmed utilizing the same technology, with a 100% concordance rate (95% confidence interval [CI]: 66.4%–100.0%).

The complete lack of recombination was confirmed in the re-biopsy of one embryo. Re-biopsy of one embryo with an average number of recombination events confirmed the presence of recombination, although with a slight difference (16 windows in the clinical biopsy vs. 21 windows in the re-biopsy). Finally, the performance of our bespoke genotyping tools used in PGT was compared to SNP-array results obtained in re-biopsies of five embryos (Figure S5). Triploidy, isodiploidy, and their respective meiotic origins were confirmed in all embryos by means of a SNP array. Recombination events detected using the SNP array in triploid embryos mapped within the recombination windows detected using PGT in 37 out of 38 events (97.4%; 95% CI: 86.2%–99.9%) with a probability of it being due to random chance of *p* < 0.001 (permutation test).

## Results

### Incidence of ploidy abnormalities in ICSI-generated blastocysts

**Figure 1:**
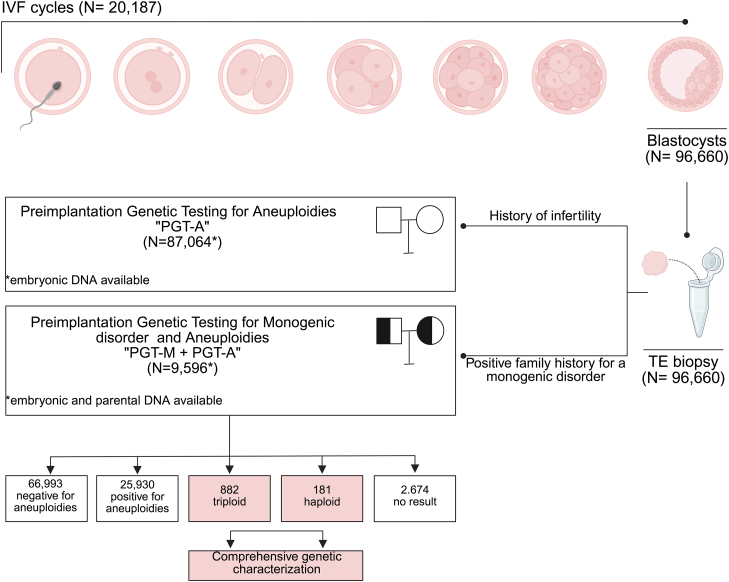
Summary of the main dataset and clinical workflow A total of 96,660 blastocyst-stage embryos, derived from 20,187 IVF cycles with apparently normal fertilization, underwent trophectoderm biopsy for preimplantation genetic testing. Among these, 87,064 TE biopsies were of embryos from couples with infertility, and PGT was intended to detect aneuploidy (PGT-A). Conversely, in 9,596 TE biopsies from couples with a positive family history for a monogenic disorder, PGT was performed for both aneuploidy and the specific monogenic disorder (PGT-M). In this subgroup, both embryonic and parental DNA were available for genetic analysis. Comprehensive genetic characterization was performed on triploid and haploid samples.

To determine the incidence of ploidy abnormalities in human preimplantation embryos, we used data obtained from 96,660 trophectoderm biopsies (dataset A; see subjects, material, and methods and Table S1) from 20,187 IVF cycles that used a targeted NGS protocol for PGT for aneuploidy (PGT-A) or monogenic (PGT-M) disorders (Figure 1). All embryos generated using ICSI and 2PNs were identified after fertilization during the routine, static, morphological assessment performed by the embryologists. 93,986 embryos provided high-quality informative samples, of which 66,993 embryos were euploid (71.3%; 95% CI: 71.0%–71.6%) and 25,930 were aneuploid (27.6%; 95% CI: 27.3%–27.9%) (Figure 1). As expected, the aneuploidy rate was positively correlated with advanced maternal age, regardless of the indication for testing (PGT-A or PGT-M) (Figure S1).

**Figure 2:**
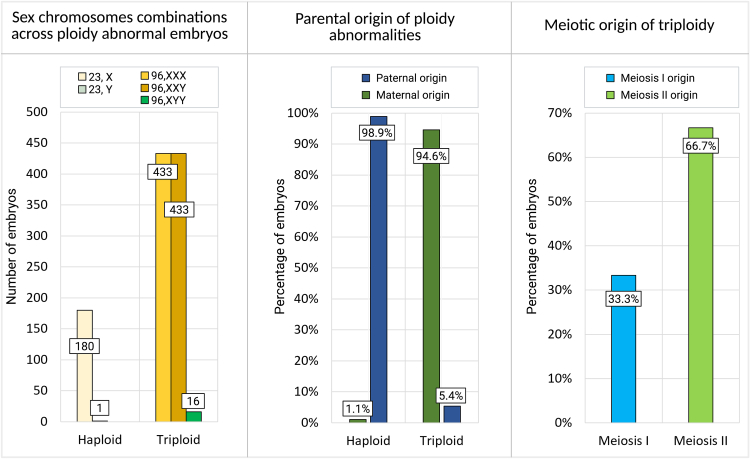
Summary of the ploidy of abnormal ICSI-derived embryos From left to right: (i) histogram depicting the distribution of different sex chromosome combinations observed in haploid and triploid embryos; (ii) histogram showing the estimated percentage of haploid embryos, categorized by whether the chromosomal abnormality originated maternally or paternally; and (iii) histogram showing the estimated meiotic origin of triploid embryos, distinguishing between segregation errors occurring in meiosis I vs. meiosis II (without differentiating between male and female meiosis).

We discovered ploidy abnormalities in 1.1% of embryos (*n* = 1,063/93,986; 95% CI: 1.06%–1.20%). Of these, 181 (17.0%; 95% CI: 14.8%–19.4%) were found to be haploid (Figures 1 and 2). Only one haploid embryo had a Y chromosome, while the X chromosome was present in all the others, demonstrating that haploidy is almost entirely representative of paternal origin, most likely due to a missed extrusion or decondensation of sperm DNA following ICSI (*p* < 2.2 × 10^−16^; binomial exact test; Figure 2).

Among the 1,063 embryos with ploidy abnormalities, 882 were triploid (83.0%; 95% CI: 80.6%–85.2%; Figures 1 and 2), indicating a prevalence nearly five times greater compared to haploid abnormalities in blastocysts. Among the triploid embryos, 433 had three X chromosomes (69, XXX; 49.1%), 433 embryos contained two X chromosomes and one Y (69, XXY; 49.1%), and 16 embryos had one X chromosome and two Y (69, XYY; 1.8%) (Figure 2).

These observations suggest a 4.8-fold preponderance of triploid over haploid blastocysts (882 compared to 181). Thus, the excess of triploid fetal losses compared to haploid ones in prenatal diagnosis is due not only to selection during fetal development but also to a bias already present at the blastocyst stage.

### Parental and meiotic phase of origin of ploidy abnormalities

Next, we investigated the parental contribution and meiotic origin of ploidy errors in the 1,063 embryos that contained ploidy abnormalities. We first used a modeling approach, based on the sex chromosome constellation (XXX/XXY/XYY), and assumed independence between parental and meiotic origins. We estimated that in 94.6% of triploid embryos, the extra chromosome set was of maternal origin, whereas 5.4% were of paternal origin (Figure 2). Triploid embryos arose from a failure to segregate chromosomes both in the first and second meiotic divisions (33.3% and 66.7%, respectively; Figure 2). In contrast, 98.9% of the 181 haploid embryos were due to a missing set of paternal chromosomes, based on having only a single haploid embryo with a Y chromosome (Figure 2).

To directly determine the parent of origin and meiotic phase at which entire chromosome sets failed to segregate, we used SNP genotyping data from embryos and parents (“trios”) undergoing testing for monogenic disorders. Of the 55 trios analyzed, haploid embryos were confirmed to be predominantly caused by an absence of paternal genome (12 of 14; 85.7%; 95% CI: 57.2%–98.2%). All 41 triploid embryos (100%; 95% CI: 91.4%–100.0%) were due to female meiosis errors.

Meiotic recombination is reduced near centromeres, and informative SNPs (within 5 cM from the centromere) can be used to detect the presence of homologs (MI) vs. sister chromatids (MII) in triploid embryos. Eleven of the 41 triploid embryos (26.8%; 95% CI: 14.2%–42.9%) had both maternal homologs for all chromosomes and were therefore caused by genome-wide segregation failure in MI. The remaining embryos carried alleles from only a single homolog around all centromeres and were inferred to originate from genome-wide failure to segregate sister chromatids at MII (*n* = 30/41; 73.2%; 95% CI: 57.1%–85.8%). Triploid conceptions that originate from maternal meiosis are also found in natural conceptions,^11^^,^^30^^,^^31^ suggesting that these observed effects are not due to ovarian stimulation.

### Recombination rates of triploid embryos

To investigate a potential causal relationship between ploidy abnormalities and recombination events, we analyzed SNP inheritance patterns for the 41 triploid embryos for which parental genotyping data were available. Meiotic recombination between homologous chromosomes caused switches between regions of SPHs or BPHs along chromosomes. We detected the switches using the delta inconsistency score (subjects, material, and methods) after correcting for the number of usable SNPs (Table S2; Figure S2).

**Figure 3:**
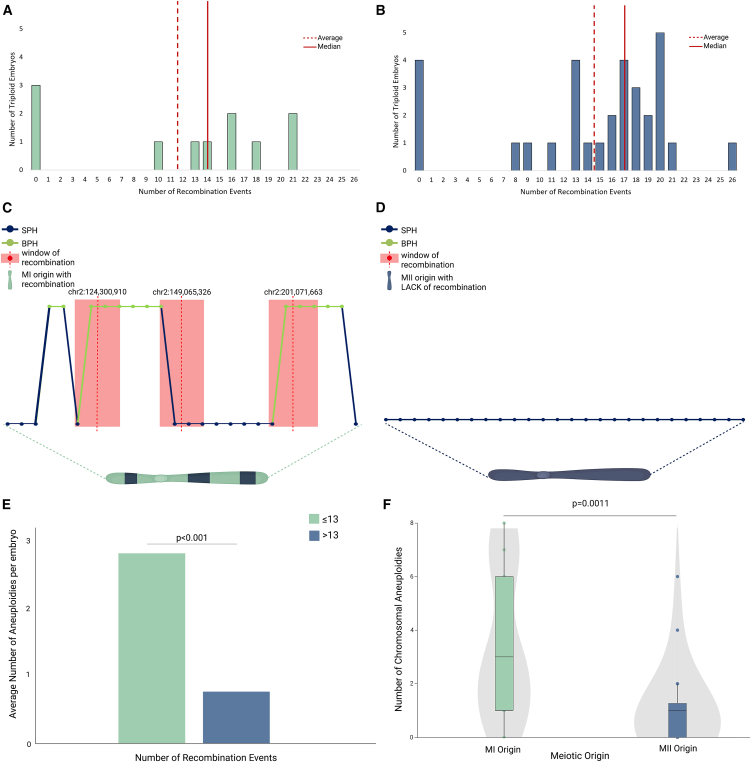
Recombination events in triploid embryos and their association with aneuploidy rate (A and B) Bar plots depicting recombination events in triploid embryos of meiosis I (MI) origin (A) and meiosis II (MII) origin (B). (C and D) Graphic representation of recombination windows detected in chromosome 2 from an embryo with an average number of recombination events and MI origin (C) and an embryo with a lack of genome-wide recombination and MII origin (D). Each dot represents the delta score for each SNP, with green indicating a BPH state for maternal alleles from an MI error and blue indicating an SPH state for maternal alleles from an MII error. The red dot corresponds to a point of switch of the average score. The red rectangle highlights the window of recombination described as the area within the identified point of switch and the closest preceding SNPs with an opposite average score value. (E) Bar plot illustrating a statistically significant correlation between the average number of aneuploidies per embryo and recombination rates, comparing one group of embryos with low recombination rates (<13 events) and a second group with higher recombination rate (>13 events); 13 is the mean number of recombination events in our dataset. (F) Boxplot and violin plots showing a different rate of additional aneuploidies in triploid embryos of MI origin (green) and MII origin (blue).

The number of detectable recombination events ranged from 0 to 26 in the triploid embryos of maternal origin (Figures 3A and 3B; Table S2). Of these, most contained between 8 and 26 recombination events genome wide (Figure 3C). This number of recombination events is lower than the 41–46 previously observed in human preimplantation embryos^32^^,^^33^; in particular, the number of SPH/BPH switches is higher than the number of recombination events that would have been observed in a diploid embryo (Note S1). This discrepancy may serve as a potential biomarker for gross chromosomal abnormalities. However, a possible bias may result from differences between the two technologies, as some crossovers may be missed due to the sparse genome coverage of our assay (e.g., Figure 3C).

Seven of the triploid embryos were inferred to lack crossovers genome wide (7/41 or 17.1%; 95% CI: 7.1%–32.1%; Figure 3D). A permutation test was employed to test if the lack of genome-wide recombination could be interpreted as an “expected” finding and revealed that these embryos constituted an outlier group (*p* < 0.001) compared to the remaining embryos. Failure to initiate meiotic recombination was inferred in diploid oocytes originating from both MI and MII (Figures 3A and 3B). Genome-wide recombination failure has previously been inferred in one human oocyte^32^ and has been shown to be associated with single chromosome aneuploidies. Since all fertilized oocytes contained a polar body for the ICSI procedure, our observations suggest that failure to segregate one chromosome set into the polar body is another consequence of genome-wide recombination failure.

We detected additional chromosomal aneuploidies in triploid embryos. This finding was statistically correlated with the meiotic origin of triploidy, with triploid embryos of MI origin being more prone to additional segregation errors (*p* < 0.001) (Figure 3F). Furthermore, a lower number of genome-wide recombination events was also found to be statistically correlated with a higher number of chromosomal aneuploidies in the triploid embryos (*p* < 0.001) (Figure 3E).

### Recombinant genome-wide uniparental isodisomy

The lack of paternal genomes in the haploid embryos and the prevalence of two maternal chromosome sets in the triploid embryos predict a class of embryos that are diploid but contain only maternal chromosome sets (maternal isodiploidy).

**Figure 4:**
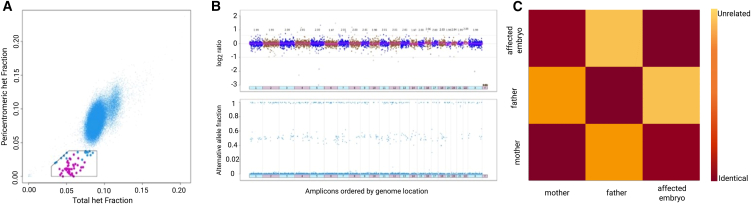
Recombinant genome-wide uniparental isodisomy in preimplantation embryos (A) Distribution of 74,009 samples according to their total het fraction (*y* axis) and pericentromeric het fraction (*x* axis). Dots in the pentagonal box represent putative isodiploid embryos, and magenta-colored dots are embryos confirmed to be isodiploid by PGT and fingerprinting plots. (B) PGT plots of an affected embryo. Top: the amplicon distribution in terms of copy number. Bottom: the alternative allele fraction, showing regions of loss of heterozygosity (absent dots with values between 0.1 and 0.9). (C) Fingerprinting plot of an affected embryo and its parents. Dark red corresponds to genetic identity, while the lighter the color, the lower the genetic correlation.

To detect this, we developed an algorithm (subjects, material, and methods) that mapped recombinant sister chromatids, genome wide, after segregation failure in MII and a normal MI. We detected 60 such events among 74,009 embryos from dataset D (see subjects, material, and methods and Table S1). Manual checking of PGT plots confirmed the finding for 41 of these (0.05%, 95% CI: 0.04%–0.07%) (Figures 4A and 4B). We refer to these as recombinant isodiploidy, and they were characterized by homozygosity in pericentromeric regions but with few detectable heterozygosity regions throughout the rest of the genome, indicating that crossovers occurred between the two parental homologs during prophase I. Additionally, fingerprinting analysis confirms a loss of genetic correlation between the affected embryo and the paternal DNA sample (Figure 4C), as well as with corresponding sibling embryos. The latter is to be expected due to the absence of one parental genome in the affected embryo compared to the rest of the sibling embryos that share genetic similarities in both the maternally and paternally inherited genomes.

### Parental age correlation

**Figure 5:**
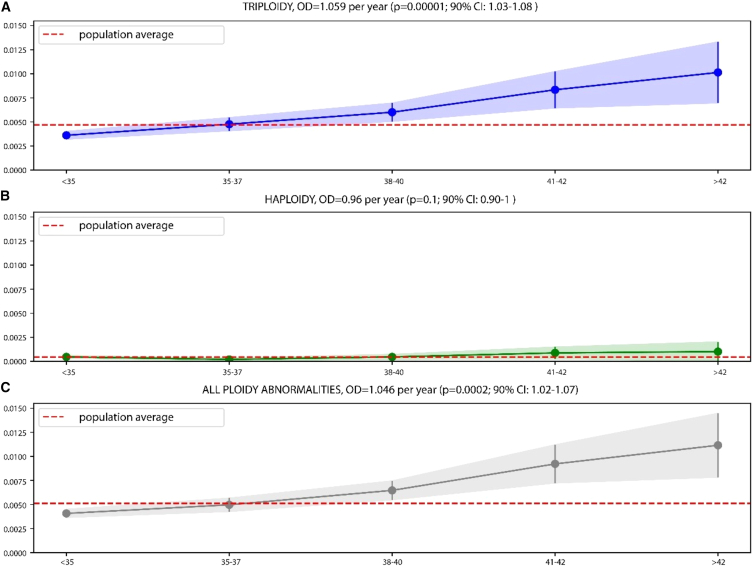
The risk of ploidy abnormalities rises with advancing maternal age (A–C) Plots illustrating the relationship between maternal age and various subsets of ploidy abnormalities. Maternal ages were classified using the SART age groups (*x* axis). The *y* axis shows the fraction of affected embryos: triploid, haploid, and all ploidy abnormalities.

The large size of our dataset, which includes 1,063 ploidy abnormalities, allowed us to correlate their incidence with parental age. The mean maternal age was 35.65 years (±2.9 years; range 20–47), and the mean paternal age was 37.22 (±3.03 years; range 19–79). We detected a positive and statistically significant correlation (OR [odds ratio] = 1.046 per year; *p* < 0.001) for ploidy abnormalities and maternal age (Figure 5). Triploidy had the strongest correlation with maternal age (OR = 1.059 per year; *p* < 0.001) (Figure 5), consistent with the finding that they have a predominant maternal origin in our dataset. The risk of having a triploid conception at female age 40 was 76% higher than at age 30.

Paternal age was significantly associated with an increased risk of triploid conceptions in the univariate analysis. However, this association disappeared in the multivariate analysis, as paternal age was linked to maternal age (Table S3).

The association between maternal age and triploidy was confirmed in an independent dataset of 44,324 embryos (dataset B; see subjects, material, and methods and Table S1), where we observed an OR of 1.03 per year (*p* = 0.03, Figure S3). We conclude that maternal age is associated with diploid oocyte formation and triploid blastocysts.

### Maternal age is associated with elevated risk of abnormal fertilization

We noted a slight difference in the effect size of female age in the two datasets, as well as a slightly lower frequency of triploidy in dataset B (0.7%; 293 of 44,324; 95% CI: 0.6–0.74; Table S1). We considered two, not mutually exclusive, explanations, including that the smaller sample size of the second dataset might explain the differences. However, a second explanation may be that the difference is caused by fewer fertilized oocytes being misclassified in the second dataset as being 2PNs, when in fact they were 3PNs, since time-lapse incubators were used. Zygotes with 3PNs are known to have a higher incidence of triploidy and may have contributed to the higher incidence in the larger dataset, where the latter did not use time-lapse incubators.^34^ This predicts that abnormal fertilization should be elevated with maternal age.

To test whether abnormal fertilization events were increased with maternal age, we used dataset C (see subjects, material, and methods and Table S1), comprising 93,341 zygotes for which the PN was available. Advanced maternal age was statistically significantly associated with an increased risk of abnormal fertilization following ICSI (1PNs and >2PNs; *p* = 1.21 × 10^7^; negative binomial regression). In particular, the presence of 3PNs had the strongest correlation with maternal age (*p* = 2 × 10^−8;^ Figure S4). Thus, the higher chance of exclusion of 3PN fertilized oocytes in the smaller validation dataset misses a subset of maternal triploid conceptions that would have arisen from abnormally pronucleated zygotes.

### Recurrence of ploidy abnormalities in a subset of individuals

**Figure 6:**
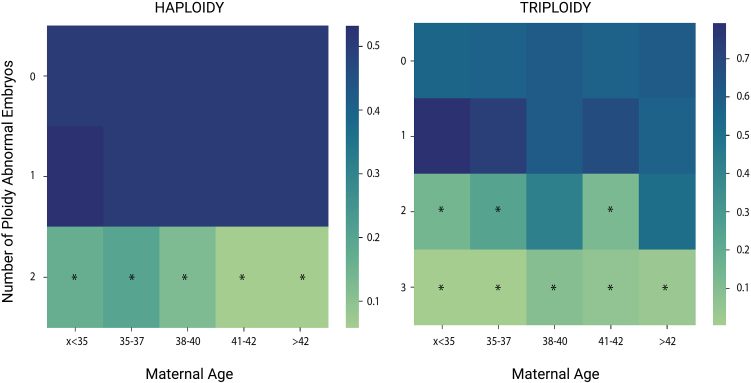
Unexpected per-family ploidy error recurrence Heatmap of Bonferroni adjusted *p* values obtained for each maternal age category (columns) and each ploidy error recurrence count (rows) for haploidy (left) and triploidy (right). *p* values represent the probability of observing the event by random chance. Heatmap squares marked with “^∗^” were considered statistically significant (*p* < 0.05; Bonferroni adjusted).

To determine whether some individuals are at an increased risk of recurrent ploidy abnormalities, we analyzed the number of affected embryos in any given treatment cycle. We used the original dataset A that consisted of 96,660 embryos obtained from 20,187 cycles and 20,187 different couples (Table S1). We found that 19,288 cycles produced no embryos with ploidy-level defects, 852 cycles produced one embryo with a ploidy-level defect (4.2%; 95% CI: 3.9%–4.5%), 41 cycles produced two (0.2%; 95% CI: 0.1%–0.3%), and six cycles produced three (0.03%; 95% CI: 0.01%–0.06%) (Table S4). In four of the six couples with three ploidy-abnormal embryos, the maternal age was <35 years. Under a null hypothesis of no family-specific causes of ploidy abnormalities, the only factor affecting the ploidy occurrence is maternal age, and embryos with ploidy abnormalities should therefore be randomly distributed among embryonic cohorts from women of similar maternal ages. However, this was not the case, as a permutation analysis showed that the recurrence of three or more ploidy-abnormal embryos from the same couple cannot be explained as a random event (Figure 6). Therefore, this analysis suggests that there may be other factors increasing the risk of ploidy abnormality recurrence.

## Discussion

In this study, we initially investigated the parental and meiotic origins of haploid and triploid blastocysts in humans. We used several independent large datasets from PGT cycles where ploidy abnormalities can be detected. The use of preimplantation human embryos derived by ICSI in infertile individuals using PGT-A and non-infertile individuals using PGT-M allowed us to indirectly infer if a gamete was affected by a ploidy abnormality. The overall incidence of ploidy abnormalities inferred as originating from gametes was 1.15%, with triploidy accounting for the vast majority (83%).^35^ These rates are similar to those reported in prenatal diagnoses of natural conceptions,^30^ when the lack of dispermy in our dataset is considered.

Some of our findings were unexpected, including that the haploid conceptions were mainly of paternal origin, indicative of an absence of DNA in the sperm head, extrusion of sperm DNA into the second polar body, or failure of the sperm chromatin to decondense at fertilization. Consistent with this, oocytes were more likely to be diploid than nulliploid, suggesting that when genome-wide chromosome segregation fails, the chromosomes are more likely to remain in the oocyte rather than segregate to the polar body. The robust datasets also allowed us to detect a maternal age effect on the generation of diploid oocytes at the levels of both MI and MII, as well as on abnormal fertilization. These effects are unlikely to be due to ovarian stimulation and ICSI, respectively, since our data show important similarities to those of *in vivo* conceptions, including the higher incidence of errors leading to triploidy in MII compared to MI.^30^^,^^36^

It has long been known that meiotic recombination may fail on individual chromosome pairs, especially the small chromosomes 21 and 22,^37^ resulting in an elevated risk of aneuploid and trisomic conception (e.g., trisomy 21). Our findings, however, reveal for the first time a process of genome-wide recombination failure that results in diploid oocytes at MI and MII. Meiotic recombination is initiated by the highly conserved Spo11-Top6BL transesterase during meiotic prophase I.^38^^,^^39^^,^^40^^,^^41^^,^^42^^,^^43^^,^^44^ Recent findings in budding yeast, however, reported that the deletion of Spo11 in some cases resulted in diploid spores, without evidence of recombination.^45^ Deletion of *Spo11* causes both male and female infertility in mice^46^^,^^47^ due to checkpoints that eliminate germ cells failing to engage in meiotic recombination, synapsis, and silencing. The meiotic-silencing checkpoint, however, is sexually dimorphic, with a less stringent response in females.^48^ Furthermore, BCL-2 (B cell lymphoma 2) has been shown to eliminate recombination-defective oocytes in mice.^49^ Our findings suggest that the initiation of meiotic recombination can fail genome wide in human fetal oocytes and that at least some of these may escape checkpoints during the fetal stages and are capable of ovulating decades later.

Failure to segregate sister chromatids during MII may be the main cause of triploid conceptions in humans. A recently published study reported a similar proportion of MI and MII errors in naturally conceived triploid conceptions,^50^ corroborating our findings in ICSI-derived IVF embryos. It was not reported whether the fetuses from pregnancy losses also displayed genome-wide recombination failure. This suggests that recombination failure may reflect an additional underlying biological defect that compromises a conceptus’s potential for survival—even in natural pregnancies.^50^ MII failure also resulted, in some rare cases, in recombinant isodiploid blastocysts characterized by diploidy in the oocyte and a lack of sperm DNA. These errors may lead to extreme imprinting disorders affecting more than 150 genes.^51^ Lastly, fertilization abnormalities and ploidy status alterations were also found to be statistically correlated with advancing maternal age, suggesting that oocytes may become more error prone with age in terms of segregating sister chromatids as well as forming 3PNs (as demonstrated when analyzing zygotes from dataset C; Table S1; Figure S5). The causes of such effects remain to be investigated. Additionally, the impact of infertility etiology on ploidy abnormalities was not investigated in this study. This was primarily due to the limited sample size, which would not have provided sufficient statistical power for meaningful subgroup analyses. While euploidy rates appear to be similar between infertile and fertile individuals, our study did not specifically assess how different infertility etiologies might affect euploidy outcomes. Although some data are available in the literature, findings remain quite discordant.^52^^,^^53^^,^^54^^,^^55^^,^^56^ Therefore, we aim to conduct future studies, with large cohorts and prospective data collection, to better evaluate the potential relationship between infertility causes and ploidy rates.

## Data and code availability

The data and codes that support the findings of this study are extensively described in the methods and available upon request to the corresponding author. Clinical data were used in this study and are not publicly available due to privacy or ethical restrictions.

## Author contributions

Conceptualization, L.P., A.C., and C.S.O.; methodology, F.M., S.C., E.M.P., L.P., C.S.O., A.C., X.T., and Y.Z.; investigation, L.P., C.S.O., M.J.E.P., L.F.R., F.M.U., and V.J.; data collection, P.G., C.M.V., M.J.E.P., and D.B.; visualization, L.P. and F.M.; supervision, C.S.O., E.R.H., A.C., J.A.G.-V., and A.P.; writing – original draft, L.P. and C.S.O.; writing – review & editing, L.P., C.S.O., E.R.H., A.C., S.C., and C.W.

## Declaration of interests

L.P., C.S.O., F.M., and E.M.P. are employee at Juno Genetics, Rome, Italy, a company performing reproductive genetics. X.T., Y.Z., V.J., and E.M. are employee at Juno Genetics, Basking Ridge, New Jersey, USA, a lab performing preimplantation genetic testing. A.C. is chief scientific officer of Juno Genetics, Rome, Italy. D.B. is a full-time employee at Juno Genetics, Oxford, UK, a lab performing preimplantation genetic testing. L.F.R. reports personal fees from Merck KGaA, MSD, Ferring, IBSA, Cooper Surgical, Cook, Medea, Nterilizer, and Fujifilm-Irvine Scientific, outside the submitted work. C.W. is a full-time employee of IVIRMA, Clinical Research, Basking Ridge, New Jersey. S.C. reports receiving personal fees from MyHeritage outside the submitted work and was supported by the National Human Genome Research Institute of the National Institutes of Health (R01HG011711). C.M.V. is an employee at Juno Genetics, Valencia, Spain, a lab performing preimplantation genetic testing.

## Web resources

SART National Summary Report, https://www.sartcorsonline.com/rptCSR_PublicMultYear.aspx

## Supplemental information

Document S1. Figures S1–S5, Tables S1–S7, and Note S1

Document S2. Article plus supplemental information
