# Supplementary material for: Maternal age and genome-wide failure of meiotic recombination are associated with triploid conceptions in humans
Source: Am J Hum Genet. 2025 Oct 14;112(11):2665–78. doi: 10.1016/j.ajhg.2025.09.014 (PMC12808983; doi:10.1016/j.ajhg.2025.09.014)

The American Journal of Human Genetics, Volume *112*

Supplemental information

Maternal age and genome-wide failure of meiotic recombination are associated with triploid conceptions in humans

Ludovica Picchetta, Christian Simon Ottolini, Xin Tao, Yiping Zhan, Vaidehi Jobanputra, Carlos Marin Vallejo, Francesca Mulas, Elvezia Maria Paraboschi, Maria José Escribá Pérez, Thomas Molinaro, Christine Whitehead, Pavan Gill, Emily Mounts, Dhruti Babariya, Laura Francesca Rienzi, Filippo Maria Ubaldi, Juan Antonio Garcia-Velasco, Antonio Pellicer, Shai Carmi, Eva R. Hoffmann, and Antonio Capalbo

SUPPLEMENT

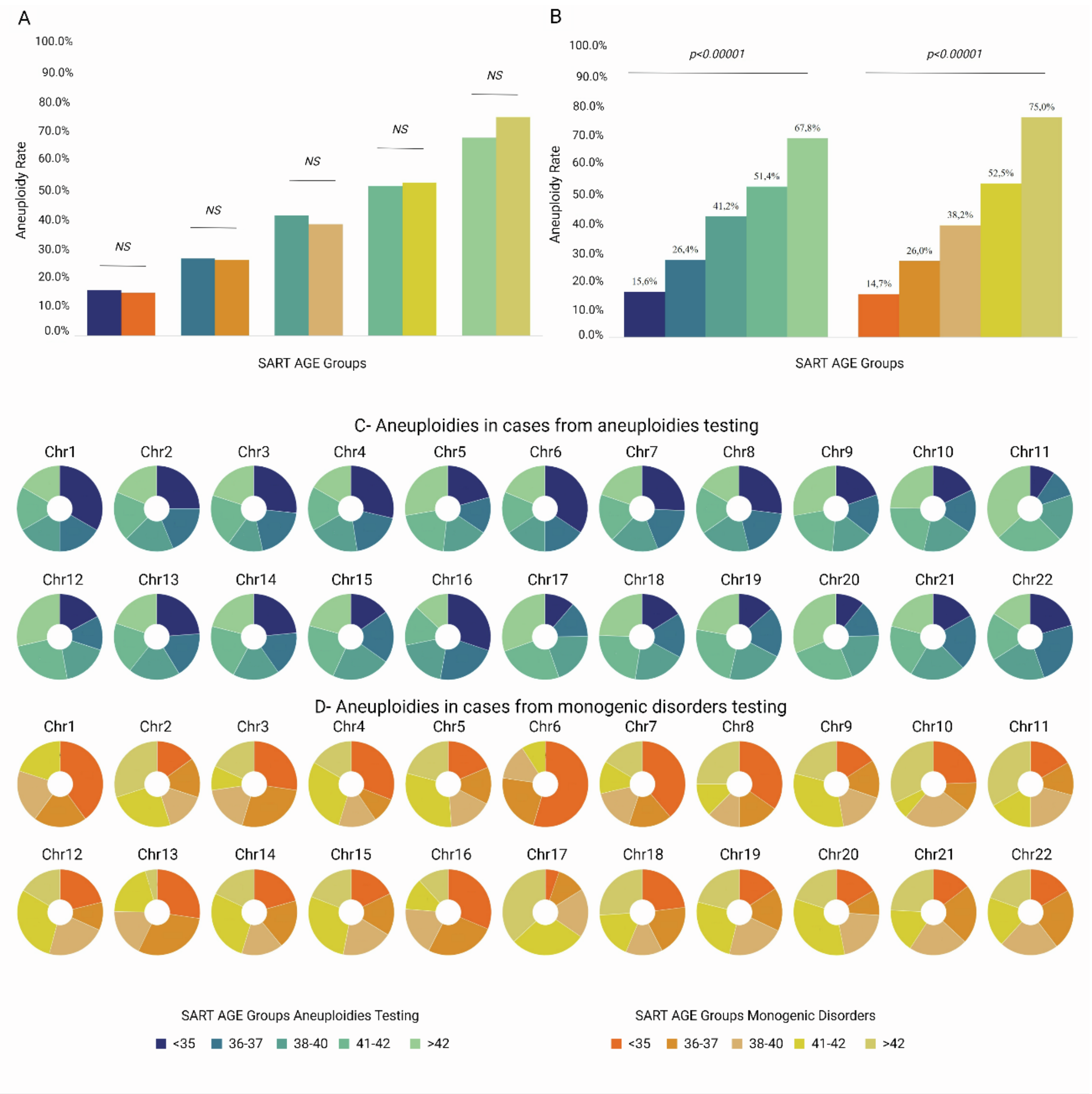

Figure S1. Impact of maternal age on aneuploidy rates across preimplantation genetic testing indications for datasets A and B. A and B- Histograms depicting aneuploidy rates in the 5 SART age groups. No statistical differences were detected amongst the different SART age groups when comparing indication for testing. However, both groups demonstrated a statistically significant correlation between advanced maternal age and increased aneuploidy rates. C-D Pie charts showing the frequencies of aneuploidies (both trisomies and monosomies) for each autosomal chromosome according to SART age group.

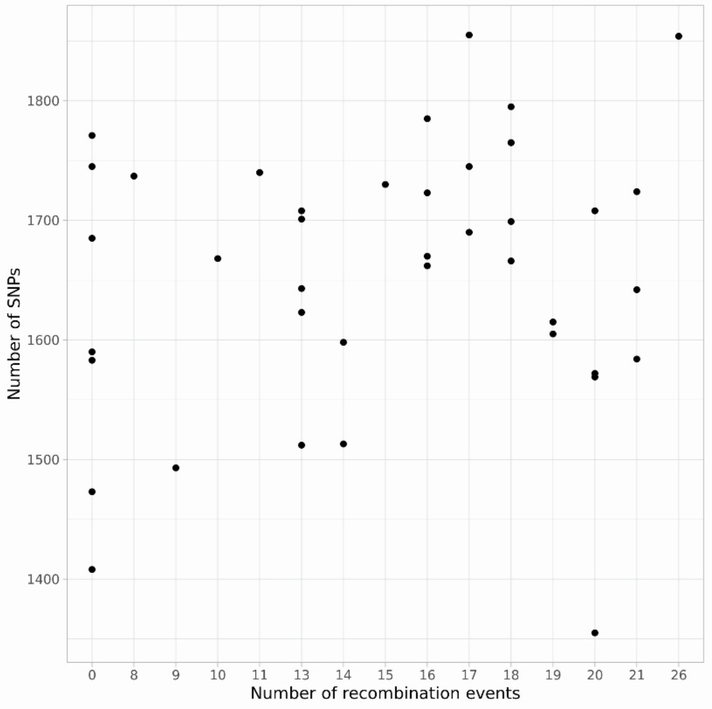

Figure S2. Distribution of the number of SNPs (y axis) vs the number of recombination events (x axis).

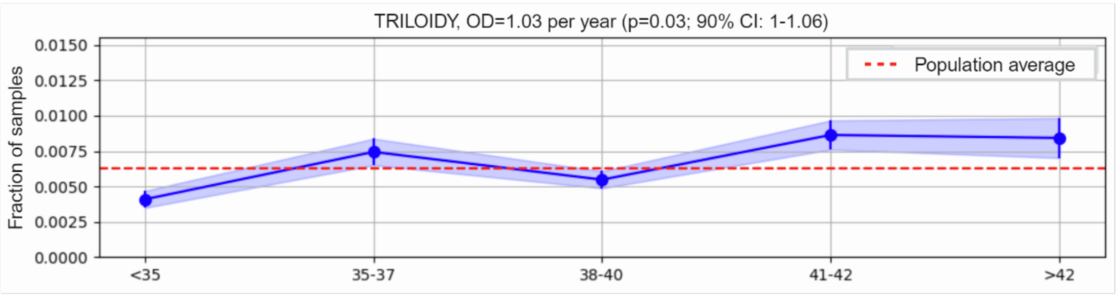

Figure S3. The risk of ploidy abnormalities rises with advancing maternal age: confirmation from an independent dataset. Plot illustrating the relationship between maternal age and triploidy in an independent dataset. Maternal ages were classified using the SART age groups (X axis). The Y axis shows the fraction of affected triploid embryos.

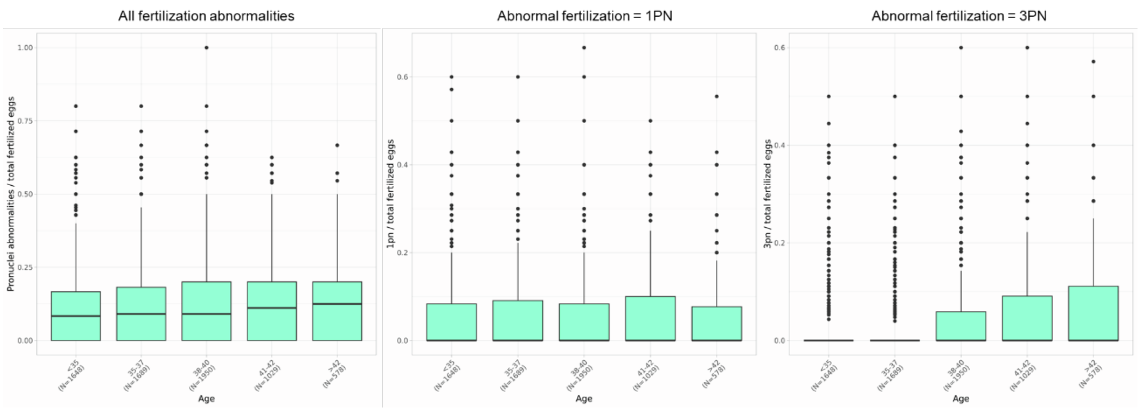

Figure S4. Maternal age is correlated with an increased risk of abnormal fertilization. Box plots representing, from left to right: distribution of (i) all abnormally fertilized oocytes, (ii) of oocytes with 1 pronucleus and (iii) of oocytes with 3 pronuclei, against female age groups. Only cycles with more than 5 inseminated oocytes are plotted.

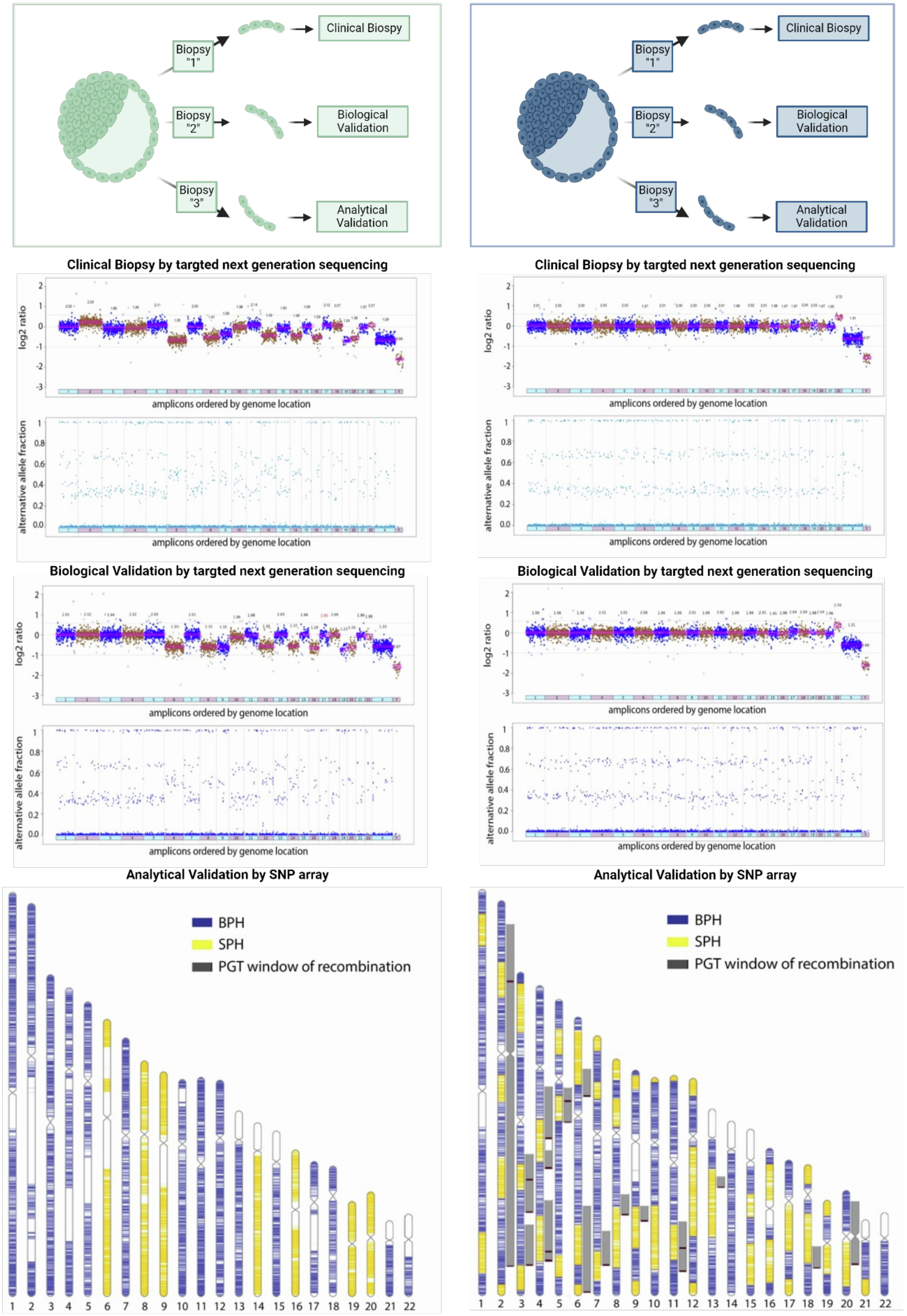

Figure S5. Reproducibility of the methods and concordance with SNP-array. This figure illustrates the multi-biopsy model, where three consecutive trophectoderm biopsies were taken from a single embryo. The first biopsy

served as a clinical sample for preimplantation genetic testing (PGT), the second for biological validation, and the third for analytical validation using SNP-array analysis. The left panel depicts an embryo with lack of recombination, meiosis I origin and additional aneuploidies. The panel on the right depicts an embryo with average recombination rate, meiosis I origin of triploidy and one additional aneuploidy on chromosome 22. The first two plots (top to bottom) show the concordance between the clinical biopsy (1st Biopsy) and the rebiopsy (2nd Biopsy) in terms of ploidy level abnormalities and additional aneuploidies. The last plot shows ideograms of the maternally inherited chromosomes distribution of SPH (yellow) and BPH (blue) SNPs according to SNP-array. The white areas on the chromosome ideograms indicate regions with no SNP coverage. Grey bars adjacent to the ideograms (separated by small black bars where two grey bars meet at the same genomic region) depict PGT windows of recombination, within which the switches from BPH to SPH were identified.

DATASET ID	# samples	Brief description	Objective
DATASET A	96,660	Embryo biopsies analyzed by targeted NGS from 2PN derived blastocyst stage embryos from ICSI cycles from IVF clinics located in the US	Main dataset employed for the investigation of haploidy, triploidy, and related biological/clinical factors in human embryos
DATASET B	44,324	Embryo biopsies analyzed by targeted NGS from blastocyst stage embryos from ICSI cycles from IVF clinics located in Europe	Validation dataset employed to corroborate findings related to triploidy and haploidy from DATASET A
DATASET C	93,341	Zygotes obtained following ICSI with annotation of the number of pronuclei to assess fertilization	Validation dataset employed for the investigation of the relationship between maternal age and abnormal fertilization in human embryos
DATASET D	74,009	Female embryos biopsies analyzed by targeted NGS from blastocyst stage embryos from ICSI cycles from IVF clinics located in US	Selected female embryos from an expanded version of DATASET A employed for the investigation of recombinant isodiploidy in human embryos

Table S1. Description of the four datasets used in the study.

PGT-M_SAMPLE	NUMBER OF USABLE INFORMATIVE SNPs	RECOMBINATION EVENTS
1	1771	0
2	1745	0
3	1590	0
4	1583	0
5	1685	0
6	1473	0
7	1408	0
8	1737	8
9	1493	9
10	1668	10
11	1740	11
12	1708	13
13	1643	13
14	1701	13

15	1512	13
16	1623	13
17	1598	14
18	1513	14
19	1730	15
20	1785	16
21	1662	16
22	1723	16
23	1670	16
24	1745	17
25	1855	17
26	1690	17
27	1765	18
28	1765	18
29	1795	18
30	1666	18
31	1699	18
32	1615	19
33	1605	19
34	1708	20
35	1569	20
36	1355	20
37	1572	20
38	1584	21
39	1724	21
40	1642	21
41	1854	26

Table S2. Number of usable SNPs and detectable recombination events per each triploid embryo in the research pipeline.

Age groups	B	S.E.	p value	OR	95% CI per OR	
paternal.age <35 yo			0,587			
paternal.age 35-37 yo	-0,176	0,114	0,122	0,838	0,67	1,048
paternal.age 38-40 yo	-0,156	0,125	0,212	0,856	0,67	1,093
paternal.age.group 41-42 yo	-0,134	0,155	0,385	0,874	0,646	1,184
paternal.age.group > 42 yo	-0,17	0,138	0,218	0,844	0,644	1,106
maternal.age <35 yo						
maternal.age.group 35-37 yo	0,414	0,119	0,001	1,512	1,197	1,91
maternal.age.group 38-40 yo	0,503	0,118	0,000	1,654	1,311	2,085
maternal.age.group 41-42 yo	0,873	0,14	0,000	2,394	1,818	3,152
maternal.age.group > 42 yo	0,804	0,151	0,000	2,234	1,663	3,001

Table S3. Paternal age is not correlated to embryonic ploidy, even when corrected for maternal age (significant P values are highlighted in red).

CoupleID_Embryo	Female Age Grouped	Male Age Grouped	Ploidy level
1_1	<35	35-37	diploid
1_2	<35	35-37	diploid
1_3	<35	35-37	triploid
1_4	<35	35-37	haploid
1_5	<35	35-37	diploid

1_6	<35	35-37	diploid
1_7	<35	35-37	diploid
1_8	<35	35-37	diploid
1_9	<35	35-37	triploid
2_1	<35	<35	diploid
2_2	<35	<35	triploid
2_3	<35	<35	triploid
2_4	<35	<35	triploid
3_1	41-42	38-40	triploid
3_2	41-42	38-40	diploid
3_3	41-42	38-40	diploid
3_4	41-42	38-40	diploid
3_5	41-42	38-40	triploid
3_6	41-42	38-40	diploid
3_7	41-42	38-40	triploid
4_1	<35	<35	diploid
4_2	<35	<35	diploid
4_3	<35	<35	diploid
4_4	<35	<35	triploid
4_5	<35	<35	triploid
4_6	<35	<35	diploid
4_7	<35	<35	triploid
4_8	<35	<35	diploid
5_1	<35	<35	triploid
5_2	<35	<35	triploid

5_3	<35	<35	triploid
6_1	35-37	35-37	diploid
6_2	35-37	35-37	triploid
6_3	35-37	35-37	triploid
6_4	35-37	35-37	diploid
6_5	35-37	35-37	diploid
6_6	35-37	35-37	diploid
6_7	35-37	35-37	triploid

Table S4. Ploidy abnormalities recurrence (≥3). Six couples were found to have a ploidy abnormality recurrence within a single IVF/ICSI cycle.

Female genotype	Male genotype	Embryo genotype	Probability BPH	Probability SPH	Inconsistency BPH	Inconsistency SPH
AB	AA	AAA	0	1	1	0
AB	AA	ABB	0	1	1	0
AB	AA	AAB	1	0	0	1
AB	BB	BBB	0	1	1	0
AB	BB	AAB	0	1	1	0
AB	BB	ABB	1	0	0	1

Table S5. Examples explicative of the scoring system used for probabilities and inconsistencies of BPH and SPH. Given specific allelic combination in the parents, for each embryonic genotype a probability of it BPH or SPH was

assigned. Subsequently, each probability equal to 0 and 1 was converted into an inconsistency score of 1 or 0, respectively.

Female genotype	Male genotype	Obligated embryonic genotype	Inconsistent embryonic genotypes	Meaning
AA	BB	AAB or ABB (hetero)	AAA and BBB (homo)	Allele Drop Out (ADO)
BB	AA	BBA or BAA (hetero)	AAA and BBB (homo)	Allele Drop Out (ADO)
AA	AA	AAA (homo)	AAB or ABB (hetero)	Allele Drop In (ADI)
BB	BB	BBB (homo)	AAB or ABB (hetero)	Allele Drop In (ADI)

Table S6. Evaluation of genotyping error rate in triploid embryos. Given specific allelic combination in the parents, embryonic genotype at that locus had to be either heterozygous or homozygous. If embryonic genotype was inconsistent due to allele drop out or allele drop in mechanisms, this was interpreted as indication of a SNP genotyping error.

	Biological validation (1st Rebiopsy)			Analytical validation (2nd Rebiopsy)	
Original PGT Results	Re-biopsy for confirmation of ploidy abnormality using PGT	Re-biopsy for meiotic origin and recombination using PGT	Origin	Re-biopsy using SNP-array	Origin
Triploid	yes	yes	TE	yes	TE
Triploid	yes	yes	TE	yes	TE
Triploid	yes	NA	ICM	yes	TE
Triploid	yes	NA	ICM	NA	-

Triploid	yes	NA	ICM	NA	-
Triploid	yes	NA	ICM	NA	-
Triploid	yes	NA	ICM	NA	-
Isodiploid	yes	NA	TE	yes	TE
Isodiploid	yes	NA	TE	yes	TE

Table S7. Schematic summary of biological and analytical validation of the genotyping tools.

Note S1

A method to infer the total map length considering that the proportion of chiasmata that are visible as a crossover is different between the MI-error-like state (both parental homologs) and MII-error-like state (same parental homolog).

As shown in the illustration at the end of this text, any chiasma in a single parental homolog (SPH) segment will change the state to both parental homolog (BPH). In contrast, only half of the chiasmata in BPH segments will be observed as changing the state to SPH. We assume no crossover interference and no chromatid interference. (Such that each chiasma has an independent probability of ½ to be observed.)

Denote the chiasmata rate along the genome (say, per bp) as λ. We assume the same rate regardless of whether the state is SPH or BPH.

For an SPH segment of length x, the probability of no event up to length x and then transition to BPH is $\lambda e^{-\lambda x}$.

For a BPH segment of length x, the probability of no event up to length x and then transition to SPH is $\frac{\lambda}{2} e^{-\lambda x/2}$. This is because the probability of a transition (in each infinitesimal length unit) is λ for a chiasma, and then ½ for the chiasma to be observed.

Suppose we have L_s bp in SPH state and L_b in BPH, with $L = L_s + L_b$. Denote the number of events at the end of SPH and BPH segments as n_s and n_b, respectively, and $n = n_s + n_b$.

The likelihood of the data is thus: $\lambda^{n_s} e^{-\lambda L_s} \left(\frac{\lambda}{2}\right)^{n_b} e^{-\lambda L_b/2} = \left(\frac{1}{2}\right)^{n_b} \lambda^n e^{-\lambda(L_s + L_b/2)}$.

The log likelihood is (up to factors independent of λ) $n \log \lambda - \lambda(L_s + L_b/2)$.

Taking the derivative with respect to λ and equating to zero gives an equation for the maximum likelihood estimator,

$\frac{n}{\hat{\lambda}} - (L_s + L_b/2) = 0$ or $\hat{\lambda} = \frac{n}{L_s + L_b/2}$.

This gives an estimate for the rate in which chiasmata are generated along the genome. The estimated total number of chiasmata genome-wide is $\hat{\lambda} L$, and

the estimated total map length, denoted $\widehat{M}$, is $\widehat{M} = \hat{\lambda}L/2$, because only half the chiasmata become crossovers. The expected number of crossovers genome-wide is the total map length.

We thus have $\widehat{M} = \frac{\hat{\lambda}L}{2} = \frac{nL}{2(L_s+L_b/2)} = \frac{n}{\frac{2L_s+L_b}{L}}$.

Finally, denote by $\alpha = L_s/L$ the proportion of the genome in SPH segments. Accordingly, $L_b/L = 1 - \alpha$. We thus have $\widehat{M} = \frac{n}{\frac{2L_s+L_b}{L}} = \frac{n}{2\alpha+(1-\alpha)} = \frac{n}{1+\alpha}$.

This makes sense, because, for example, if $\alpha \ll 1$ (almost all BPH), we have $\widehat{M} \approx n$, because we observe only half the chiasmata to begin with, which is the expected number of crossovers. If $\alpha \approx 1$ (almost all SPH), we have $\widehat{M} \approx n/2$, because we observed every single chiasmata, so we must divide by 2 to get the number of crossovers.

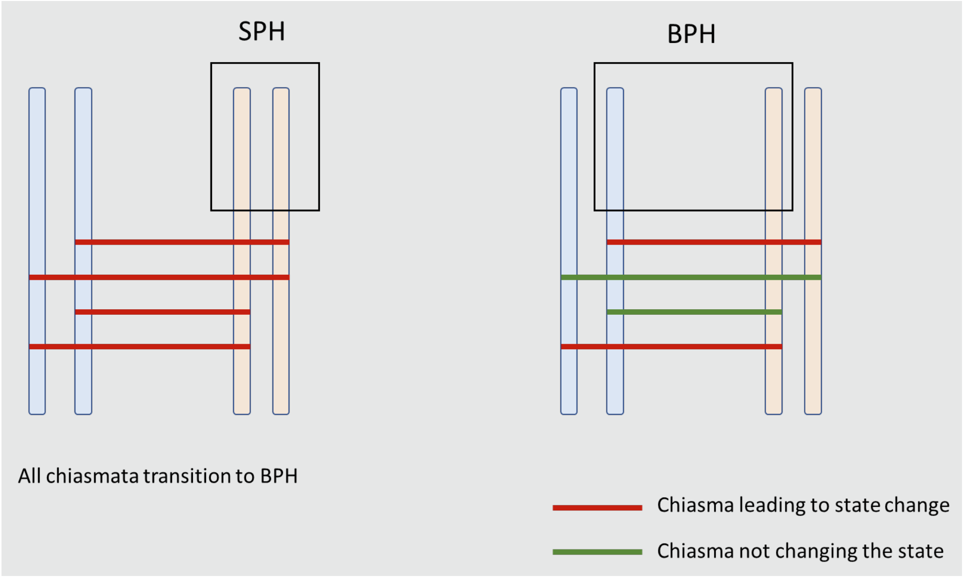

Supplement: Document S1. Figures S1–S5, Tables S1–S7, and Note S1 [file mmc1.pdf]
